# Adaptive Grasping of Moving Objects through Tactile Sensing

**DOI:** 10.3390/s21248339

**Published:** 2021-12-14

**Authors:** Patrick Lynch, Michael F. Cullinan, Conor McGinn

**Affiliations:** School of Engineering, Trinity College Dublin, D02 PN40 Dublin, Ireland; cullinmf@tcd.ie (M.F.C.); mcginnc@tcd.ie (C.M.)

**Keywords:** tactile sensing, reactive control, robotic grasping, adaptive control, grasping, dynamic grasping

## Abstract

A robot’s ability to grasp moving objects depends on the availability of real-time sensor data in both the far-field and near-field of the gripper. This research investigates the potential contribution of tactile sensing to a task of grasping an object in motion. It was hypothesised that combining tactile sensor data with a reactive grasping strategy could improve its robustness to prediction errors, leading to a better, more adaptive performance. Using a two-finger gripper, we evaluated the performance of two algorithms to grasp a ball rolling on a horizontal plane at a range of speeds and gripper contact points. The first approach involved an adaptive grasping strategy initiated by tactile sensors in the fingers. The second strategy initiated the grasp based on a prediction of the position of the object relative to the gripper, and provided a proxy to a vision-based object tracking system. It was found that the integration of tactile sensor feedback resulted in a higher observed grasp robustness, especially when the gripper–ball contact point was displaced from the centre of the gripper. These findings demonstrate the performance gains that can be attained by incorporating near-field sensor data into the grasp strategy and motivate further research on how this strategy might be expanded for use in different manipulator designs and in more complex grasp scenarios.

## 1. Introduction

Robotic manipulators are widely deployed to grasp and manipulate objects in controlled environments, such as manufacturing plants and factory assembly lines, where the structured nature of the environment can be leveraged to reduce the complexity of the manipulation task. However, for them to be effective in a wider range of settings, including in collaborative and service robot use-cases, new methods must be developed that enable them to operate in dynamic, less structured conditions.

The ability to grasp moving objects is a challenging problem that is closely coupled with the sensing paradigm that is used. Previous work has relied heavily on far-field visual sensing, where the gripper–object intercept position and time was computed based on a real-time prediction of the object trajectory. In these control systems, estimates of the gripper–object interception point and time have been the sole inputs informing grasp initiation (i.e., when the fingers start to close), with most strategies using a predefined grasping motion. This ‘object tracking’ approach has shown some noteworthy results [1,2]; however, there are several issues that limit the applicability of these methods to real-world use-cases. Firstly, results have been achieved in a laboratory setting, where lighting and other environmental conditions were tightly controlled. Secondly, the performance has relied almost exclusively on the use of external motion capture sensors placed in the surrounding environment, which are costly and rarely available outside of research laboratories. Thirdly, due to high bandwidth requirements, accurately tracking the 3D motion of objects relative to the robot using vision sensing has required high computational effort [3,4,5].

In addition to these practical issues, there are errors associated with the use of object tracking methods to inform gripper initiation. Even with the most advanced motion tracking systems available, estimates of the exact trajectory of the moving object are subject to some degree of sensor noise and latency. Another source of error is the potential for the robot’s embodiment to become a source of object occlusion, limiting the ability of the vision sensors to track the object in the near-field of the robot [2,6]. Even assuming a perfect sensing of the object, controlling the precise location of the end effector can be affected by the propagation of small positional errors through the kinematic chain of the robot’s arm, introducing uncertainty in the exact pose of the end effector. The aggregation of these errors leads to misalignment between both the actual and optimum position and the time at which the gripper intercepts and the time at which it attempts to grasp the moving object.

It was hypothesised that the implementation of a reactive sensing approach that uses tactile sensor data could enable a more adaptive grasp that is more robust to spatial errors at the point of the gripper–object contact.

This article is structured as follows. The following section will outline relevant, prior research. Section 3 describes the experimental setup, discusses the implementation of each grasping strategy in more detail and finally describes the experimental procedure. Section 4 presents the results of testing and a subsequent statistical analysis. Section 5 examines the results, outlines findings and suggests future work. Finally, Section 6 draws conclusions from the research presented.

## 2. Prior Work

Tactile sensing is a widely used sensing modality in robotic grasping. It can help infer information about an object’s properties, such as its size, shape, weight, compliance, texture and temperature [7,8,9,10,11,12]. Furthermore, tactile sensing is essential for detecting slip between gripper and object [13], and can also be used to inform and control robotic motion, referred to as tactile servoing [14,15]. It is useful, therefore, not only in grasping tasks [16], but also in object exploration [15,17,18], object classification [8,11,19], object localisation [9], assessing grasp stability [20,21] and in-hand manipulation [22]. Despite its contribution to a large number of grasping applications and its effectiveness in unstructured environments, the contribution of tactile sensing to grasping moving objects remains unexplored.

Research on the robotic grasping of moving objects dates back to the 1990s [23], and there are numerous examples of instances where robots have been required to manipulate moving objects. Examples include grasping [24], catching [2,6,25,26], batting [27,28], juggling [29] and picking up objects on a conveyor belt [30]. Sensing for this problem can generally be broken down into two stages. In the first stage, the robot must track the far-field motion of the grasp object in order to determine the point of interception. In the second stage, the robot must sense the near-field area in proximity to the gripper to initiate and adapt the grasp.

The requirement to track the far-field trajectory of the moving object is common to all grasping approaches of this type. To our knowledge, only one study has accomplished this task using exclusively egocentric sensing, where the only sensors used to track the moving object were located on board the robot [1]. With few exceptions, object tracking has been achieved primarily by using an arrangement of cameras in the area surrounding the robot. The simplest of these exocentric sensor arrangements have involved using combinations of monocular cameras to produce stereo images, allowing for the 3D motion of the object to be inferred [3,24,31]. Researchers have also tracked the motion of moving objects using one or more structured light cameras [6,30]. In a study by Cuevas-Velasquez et al., the researchers used four structured light cameras to track the far-field motion of the object and a robot-mounted stereo camera to track the near-field motion [32]. The most sophisticated object tracking setups have used specialised motion capture hardware, designed specifically to track the motion of objects moving in a 3D space [2,25,33,34]. While these systems boast the greatest accuracy and reliability, they are expensive and require a complex environment setup.

Sensing the near-field position of the object relative to the gripper has received little attention in the literature, with most controllers initiating the grasp using only estimates of the object tracking data from surrounding cameras [2,3,6,25,31]. Some exceptions include a paper by Escaida Navarro et al., where tactile sensing in the fingers was incorporated into the strategy for grasping objects moving on a conveyor belt [30]. In another relevant study, Koyama et al. used near-field proximity sensors in a pincer gripper to dynamically adjust the gripping forces in order to catch falling objects of variable sizes and textures [34]. While both of these papers incorporated dedicated near-field sensing in the grasping strategy, the benefits of this hybrid approach over grasping strategies based only on object tracking data were not explored. A recent study by Lynch and McGinn demonstrated that a reactive control strategy using tactile sensing to initiate grasping led to significant improvements in the grasp performance, especially when the object made initial contact with the gripper towards the extremity of the finger [35]. However, since their experiments were conducted in a simulation, the transferability of these findings into the real-world is questionable, given limitations in the fidelity of robotic simulation [5,22,36,37,38]. Furthermore, it is also not clear how the same tests might be replicated using a physical testing setup.

Upon examination of the prior art, the problem of grasping moving objects remains a relatively poorly understood topic and there is a need for fundamental research to better understand how factors, such as tactile sensing, could enhance the grasping performance. Building on previous work conducted in a simulation, this research proposes a systematic way to experimentally evaluate the performance of robotic grasping algorithms tasked with grasping moving objects, and investigates how control systems that use tactile feedback can lead to an improved robustness to spatial and temporal errors in the estimated interception point when grasping an object in motion.

## 3. Materials and Methods

An experiment was formulated that involved measuring the success of a two-finger robotic gripper grasping a ball moving toward it along a horizontal plane. This provided a systematic way to test each of the parameters that were hypothesised to effect grasp success: the speed of the moving object, the position of the object–gripper contact point along the finger and the timing of the grasp (i.e., when the grasp was initiated). This experiment extends an approach previously implemented in a simulation [35] that has not been adapted for use in a real-world robotic system.

To investigate the effect tactile sensing has on the ability of a robot to grasp an object in motion, a bespoke experimental apparatus was developed. In the subsections below, key components of this apparatus are described, including: the mechanism for regulating the speed of the ball, the implementation of the 2-finger gripper and a description of the different control strategies.

### 3.1. Motion of the Ball

The moving object that was chosen for the experiment was a smooth ball (diameter = 57 mm, mass = 166 g). This object was chosen as the orientation of the ball would not affect grasp performance, its mass provided a good test of grasp effectiveness, it could be easily replicated by other researchers and its velocity could be accurately controlled using a simple inclined track.

**Figure 1:**
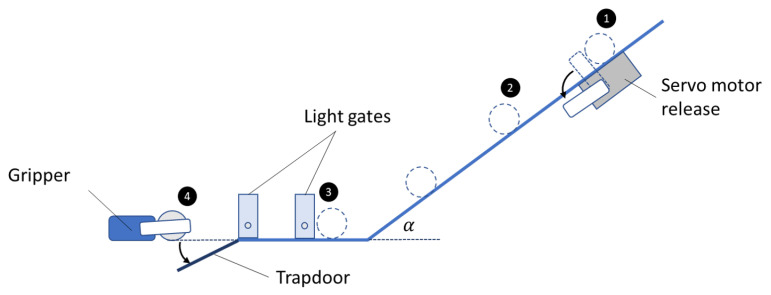
Illustration of the experimental setup. The ball is set in motion by the release of a servo arm (1). The ball rolls down the incline (2) and through the light gates, which record its velocity and notify the gripper’s controller (3). The gripper grasps (or attempts to grasp) the ball and the trapdoor is released to ensure that the full mass of the ball is supported by the gripper (4).

The ball was set in motion by allowing it to roll down an inclined plane, which was implemented using a track with a U-shaped cross-section that constrained the motion of the ball to 1 degree of freedom. At the base of the incline, the ball transitioned to a horizontal planar surface, where its speed was measured using a set of light gates. The velocity of the ball at the light gates was dependent on the release height on the inclined plane, and servo motors were used to provide a highly repeatable release mechanisms to set the ball in motion. A simple illustration of the setup is given in Figure 1. The repeatability of this approach was validated—see Table 1—two hundred tests conducted at each of the three test speeds showed a relative standard deviation of just 1.6%, 1.3% and 2.0%, respectively.

### 3.2. Implementation of 2-Finger Gripper

**Figure 2:**
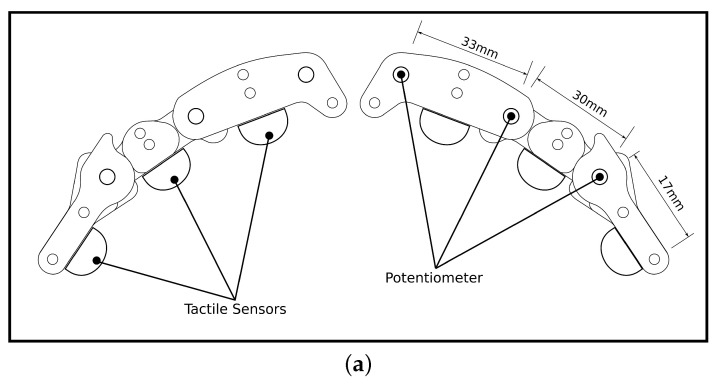

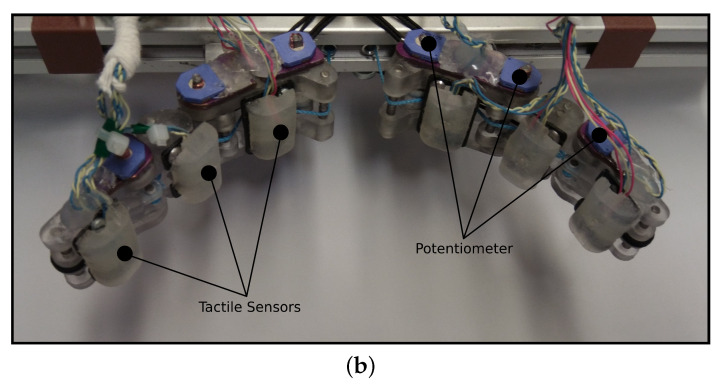
Under-actuated two-finger pincer gripper with distributed tactile sensing, shown as (**a**) diagram, (**b**) real-world gripper.

Grasping was achieved using a two-finger gripper positioned along the horizontal plane and located adjacent to the light gates. Each finger had 3 degrees of freedom and was controlled by an under-actuated cable-driven mechanism. A servo actuator was used to control closing of each finger, while the restoring action was provided by a spring connected in an antagonistic configuration. Tactile sensing was implemented using magnets embedded in the silicone fingertips (Polycraft T-15 RTV) and placed over a hall effect sensor (Adafruit MLX90393), similar to the approaches used in [39,40]. An air gap between the sensor and silicone was used to increase sensitivity, as described previously in [41]. The gripper was mounted to a servo-actuated carriage on a linear rail that ran perpendicular to the direction of motion of the ball, thus enabling the gripper to move laterally during the grasp. The layout of the gripper and a photo of the embodiment used in this experiment is given in Figure 2.

### 3.3. Grasping Strategies

Two different grasping strategies were evaluated. The first strategy involved initiating the grasp based on a prediction of when the object would contact the gripper; this approach provided a proxy to current approaches that rely solely on object tracking data to determine the grasp initiation time. The light gate was used to determine the proximity of the ball approaching the gripper; this allowed for a high resolution estimation of the position of the ball as it approached the gripper, while maintaining a lower experimental complexity compared to a vision-based object tracking system. This, in turn, allowed for different grasp timings to be tested in an accurate, repeatable way. Determination of the optimum time to initiate the grasp is complex, and subject to change with each set of experimental parameters. To contend with this, preliminary testing was conducted to approximate the optimum range of timings across the range of experimental conditions. Based on this, three grasp initiation timings were chosen, corresponding with 0 ms, 5 ms and 10 ms after passing the light gate. This procedure enabled results to be compared with the reactive strategy, with the understanding that the time offset closest to the optimum grasp initiation time will change depending on the set of experimental conditions (ball speed and location of gripper–object contact points). In this way, the optimum grasp timing, the sensitivity to deviation from this timing and comparison with the reactive strategy can be determined. Details of the implementation of the predictive strategy are given in the form of pseudocode in Algorithm 1.
**Algorithm 1:** Pseudocode describing the implementation of the predictive strategy.
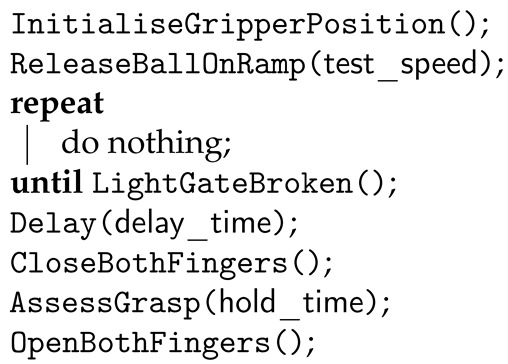


The second grasping strategy involved a reactive control system that used feedback from tactile sensing to initiate and adapt the grasping motion. This algorithm was achieved through the implementation of three basic heuristics:

1. The grasp was triggered when the object first made contact with any of the tactile sensors in the gripper;
2. Upon detection of contact with an object, the gripper moved laterally in an attempt to centre the object in the gripper.;
3. To minimise forces exerted on the ball at contact with the gripper, the closing motion of the finger that first comes into contact with the ball was delayed by 3 ms relative to the other finger. This duration was empirically determined through a process of trial and error.

Details of the implementation of the reactive strategy are give in the form of pseudocode in Algorithm 2.
**Algorithm 2:** Pseudocode describing the implementation of the reactive grasping strategy.
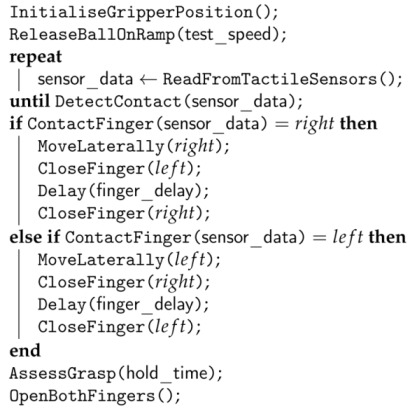


### 3.4. Experimental Procedure

For each test, the ball was placed on the incline and held in place by one of the release servos. On command, the servo released the ball, allowing it to accelerate down the incline. The ball then transitioned to the flat plane, where it passed through the light gates before the gripper would attempt to grasp it using one of the two control approaches outlined in Section 3.3. After the attempt, the platform on which the ball was rolling was lowered to remove any ground support force. The grasp was deemed a success if the gripper was able to support the full weight of the ball for 5 s.

**Figure 3:**
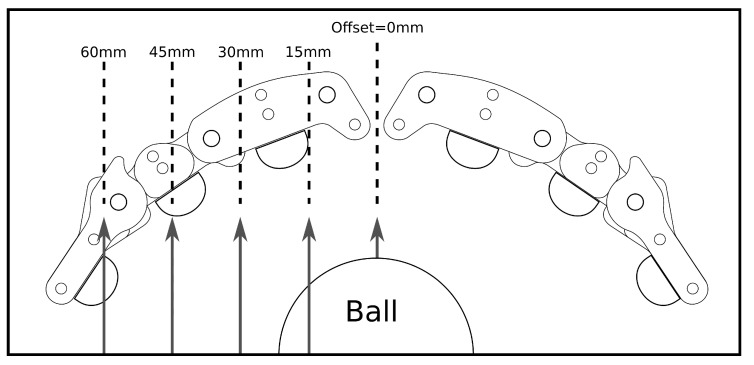
Illustration of the different gripper–object contact points that we tested in the experiment.

The robustness of the grasp to the location on the finger that the moving object made contact was tested systematically at 5 equal intervals of 15 mm (Figure 3). This was intended to replicate small positional errors in the pose of the gripper at the interception point of the object, and allowed for an examination of each strategy’s robustness to those errors. A video illustrating the testing procedure is presented in the Supplementary Materials.

## 4. Results

**Figure 4:**
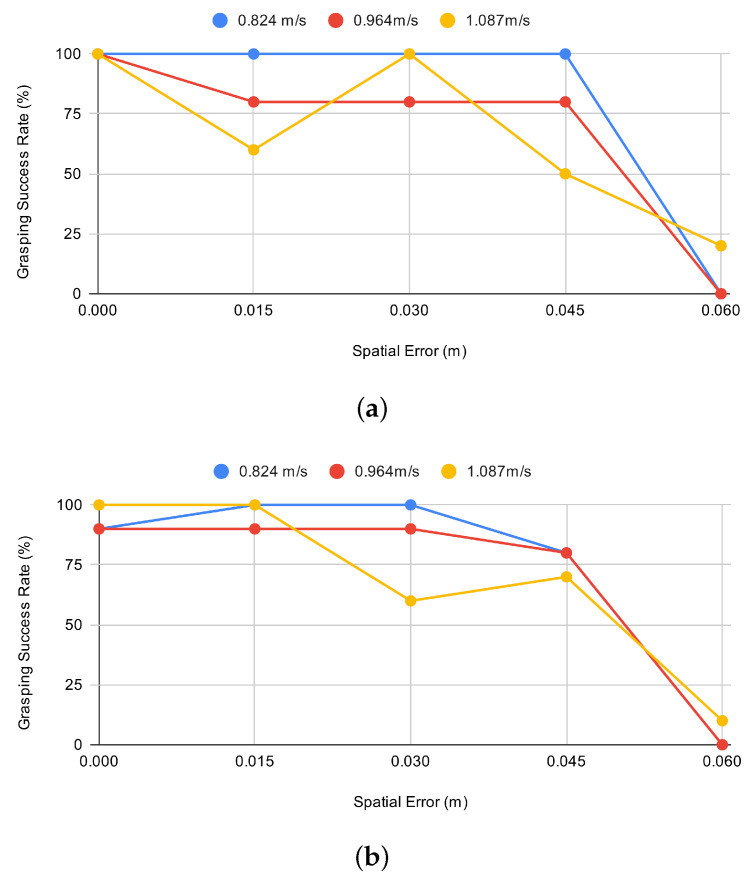

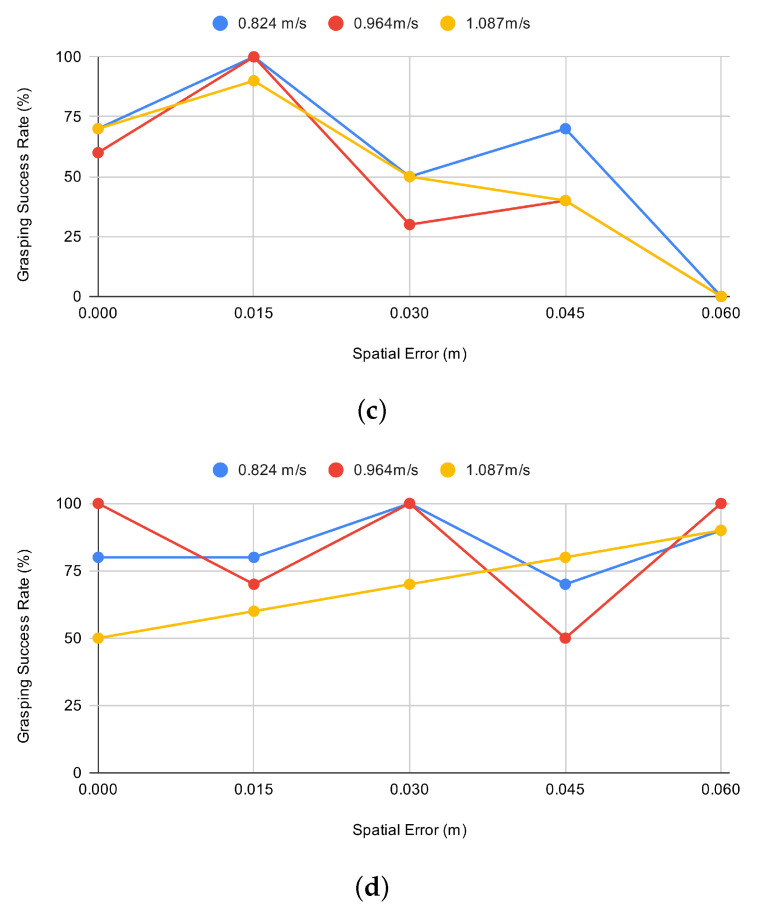
Grasping success rates at different gripper–object contact locations (spatial error) for each testing condition at three different ball speeds: (**a**) object tracking strategy with grasp initiated 0 ms after passing through timing gate; (**b**) object tracking strategy with grasp initiated 5 ms after passing through timing gate; (**c**) object tracking strategy with grasp initiated 10 ms after passing through timing gate; (**d**) reactive grasping strategy using tactile sensing.

A series of experiments were conducted to compare the performance of the reactive control strategy that used tactile sensor data to initiate the grasp with three variants of the predictive strategy that attempted the grasp based solely on anticipation of when the object would contact the gripper. The performance of each strategy was measured for different object speeds (0.82 m/s, 0.96 m/s and 1.09 m/s) and at five finger–object contact points (0 mm, 15 mm, 30 mm, 45 mm and 60 mm). Ten tests were performed for each set of conditions, with the dependent variable being if the grasp was successful or not. The grasping performance of each control strategy for each of the test conditions is illustrated in Figure 4.

To determine which controller performed best across all test conditions, data from all experiments (i.e., different ball speeds and gripper–object contact points) were aggregated for each control strategy (Table 2). From 150 grasping trials, the reactive controller that used tactile sensing achieved the best performance (79.3% success rate), followed by the predictive controller that initiated the grasp immediately after passing the light gate (71.3% success rate), 5 ms after passing the light gate (70.6% success rate) and 10 ms after passing the light gate (51.3% success rate).

For a more granular analysis, a chi-square test of independence was performed to examine the relationship] between the grasping method and grasp success for each test condition. These results are presented in Table 3.

## 5. Discussion

Results from the experiment show that the reactive strategy that initiated and adapted its grip using tactile sensing achieved the highest number of successful grasps, with a success rate of nearly 80%. The best performing predictive strategies achieved successes rates of 71.3%, 70.6% and 51.3%, respectively. The reactive controller was found to offer the greatest advantage when the gripper–object contact point was further from the centre of the gripper, where it significantly outperformed the other gripping strategies (α<0.05). These findings support earlier results obtained through performing a series of equivalent tests in a simulation [35] and verifies our original hypothesis that the implementation of a reactive sensing approach that uses tactile sensor data can enable a more adaptive grasp that is more robust to the exact point of the gripper–object contact.

Results, shown in Figure 4, show that the object tracking strategies performed best when the point of the gripper–object contact took place near to the centre of the gripper. The performance deteriorated as the gripper–object contact moved towards the extremity of the finger. These findings are consistent with results of simulation-based tests in [35] and confirm our hypothesis that grasping strategies that do not include near-field data are likely to perform poorly when the gripper is not closely aligned with the moving object.

Findings from the predictive controller suggest that the best grasp performance was achieved when the grasp was initiated as soon as the object entered its grasp envelope. This was especially true at higher object speeds, where a delayed grasp response often led to the ball bouncing off the finger on contact and subsequently escaping from the grasp. Since the reactive controller was only initiated on contact with the tactile sensor, it is likely that performance improvements in this control strategy were not due to the optimisation of the grasp initiation, but were attributable to other factors of the grasp behaviour, namely the tendency for the gripper to move laterally to centre the object in the gripper (heuristic 2) and the delay induced in the closing motion of the finger that made contact with the object (heuristic 3). This hypothesis is supported by the data because the reactive grasping strategy was found to perform best when the ball made contact with the fingertips, where the effect of these two behaviours was large, and was found to perform worse when closer to the centre of the gripper, where the behaviour of the system approximated a predictive controller with a longer grasp initiation delay. Sub-optimal grasp initiation may not have been the only factor that affected the performance of the reactive controller. With only three tactile sensors per finger, the response was not consistent for all object–finger contact locations; this issue could be addressed in future work by increasing the number of sensors in the finger, perhaps by using higher density tactile sensing technology, such as that presented in [7].

In this study, the benefits of incorporating tactile feedback in the grasping strategy was evident. However, this research also serves to highlight several avenues for future research. The testing apparatus presented enabled a systematic testing of a spherical object, where the speed and trajectory of the object could be controlled to a high degree of accuracy. Future work aims to expand upon this to enable the testing of a range of non-spherical objects, such that the orientation can also be controlled as an independent variable. Besides the shape and orientation of the target object, there are many other object parameters that could be considered in future research; these include, but are not limited to, the size, mass, texture and spin.

The tactile sensors used in this research were inspired by examples found in prior literature [41] that exhibit a high sensitivity with relatively little noise. However, future research should examine how different types and configurations of tactile sensing affects the performance. This includes, but is not limited to, different sensing mechanisms, geometry, sensing density, sensitivity and three-axis tactile sensing. There is a need to explore how the grasp performance could be further improved using near-field proximity sensing to trigger an earlier grasp initiation than is possible using tactile sensing alone. A strategy that considers time-series data (as opposed to data about the initial point of contact) could also enable further performance improvements. Machine learning techniques could improve the performance and should be investigated. Finally, this research focused on a grasping problem of a sphere rolling on a horizontal plane; however, future research could apply the same techniques to more complex grasp objects.

## 6. Conclusions

In this paper, it was hypothesised that the incorporation of a reactive control strategy that used near-field sensing to initiate and adapt a robot’s grasp could lead to a better and more robust performance for applications involving grasping moving objects. We presented the design of a testing apparatus and procedure to systematically test this hypothesis using a two-finger gripper. In our experiments, we compared the performance of a reactive grasping strategy that used tactile sensing to initiate the grasp with a traditional predictive strategy that used an estimate of the object–gripper contact point to initiate the grasp. Analysis of the data indicates that, in order to achieve the best performance, optimum grasp initiation should take place as soon as the moving object enters the gripper’s grasp envelope. Although the reactive gripping strategy initiated the grasp later than the other grasps, the effect of adaptive behaviours, such as differentially regulating the finger closure and moving the gripper laterally to centre the object within the gripper, led to it achieving the best overall grasp success rate of any of the strategies tested, with a significantly better grasping performance when the object–gripper contact point was towards the extremity of the finger. This demonstrates the importance of near-field sensing in enabling a more robust and adaptive grasping of moving objects.

## Figures and Tables

**Table 1:** The mean speed, standard deviation and standard error of the moving ball measured by the light gates for three different elevations of the incline. 200 tests were performed for each incline setting.

	Speed 1 (m/s)	Speed 2 (m/s)	Speed 3 (m/s)
Mean Object Speed	0.82363	0.96363	1.08727
Standard Deviation	0.01305	0.01252	0.02127
Standard Error	0.00092	0.00089	0.00150

**Table 2:** Summary of grasp performance across all test conditions for each control strategy, where ‘Delay’ is the delay after passing the light gate before the grasp is initiated for the ‘Predictive’ condition.

	Number of Successful Grasps			
Delay	Predictive	Reactive	$\chi^{2}$	*p*-Value
0 ms	107		2.171	0.141
5 ms	106	119	2.560	0.110
10 ms	77		24.740	0.000

**Table 3:** Summary of results from real-world grasping experiments. Where ‘Gripper Offset’ is the distance from the centre of the gripper to the point on the finger where the object makes first contact, and ‘Delay’ is the delay after passing the light gate before the grasp is initiated for the ‘Predictive’ condition. An asterisk indicates a significant difference in performance at a significance level(α)<0.05.

			Number of Successful Grasps			
Gripper Offset	Speed	Delay	Predictive	Reactive	$\chi^{2}$	*p*-Value
		0 ms	10		0.556	0.456
	0.82 m/s	5 ms	9	8	0.000	1.000
		10 ms	7		0.000	1.000
		0 ms	10		0.000	1.000
0.00 m	0.96 m/s	5 ms	9	10	0.000	1.000
		10 ms	6		2.813	0.094
		0 ms	10		4.267	0.039 *
	1.09 m/s	5 ms	10	5	4.267	0.039 *
		10 ms	7		0.208	0.648
		0 ms	10		0.556	0.456
	0.82 m/s	5 ms	10	8	0.556	0.456
		10 ms	10		0.556	0.456
		0 ms	8		0.000	1.000
0.015 m	0.96 m/s	5 ms	9	7	0.313	0.576
		10 ms	10		1.569	0.210
		0 ms	6		0.000	1.000
	1.09 m/s	5 ms	10	6	2.813	0.094
		10 ms	9		1.067	0.302
		0 ms	10		0.000	1.000
	0.82 m/s	5 ms	10	10	0.000	1.000
		10 ms	5		4.267	0.039 *
		0 ms	8		0.556	0.456
0.030 m	0.96 m/s	5 ms	9	10	0.000	1.000
		10 ms	3		7.912	0.005 *
		0 ms	10		1.569	0.210
	1.09 m/s	5 ms	6	7	0.000	1.000
		10 ms	5		0.208	0.648
		0 ms	10		1.569	0.210
	0.82 m/s	5 ms	8	7	0.000	1.000
		10 ms	7		0.000	1.000
		0 ms	8		0.879	0.348
0.045 m	0.96 m/s	5 ms	8	5	0.879	0.348
		10 ms	4		0.000	1.000
		0 ms	5		0.879	0.348
	1.09 m/s	5 ms	7	8	0.000	1.000
		10 ms	4		1.875	0.171
		0 ms	0		12.929	0.000 *
	0.82 m/s	5 ms	0	9	12.929	0.000 *
		10 ms	0		12.929	0.000 *
		0 ms	0		16.200	0.000 *
0.060 m	0.96 m/s	5 ms	0	10	16.200	0.000 *
		10 ms	0		16.200	0.000 *
		0 ms	2		7.273	0.007 *
	1.09 m/s	5 ms	1	9	9.800	0.002 *
		10 ms	0		12.929	0.000 *

## Data Availability

All data collected during this research is presented in full in this manuscript.

## Supplementary Materials

The following are available online at https://www.mdpi.com/article/10.3390/s21248339/s1, Video S1: Video Illustrating the Testing Procedure.
